# Report of the Diseases of Edinburgh for September, 1809

**Published:** 1809-11

**Authors:** John Roberton


### *Report of the Diseases of Edinburgh for September,* 1809.

By John Roberton, M. D.

ALTHOUGH, during the present month, the weather has not been so wet as during the last, yet its general tendency has been soft. On some occasions there were even very heavy falls of rain, sometimes accompanied by severe gales of wind, especially towards the end of the month, and the mornings and evenings were frequently cold and disagreeable.

The barometer, although it rose high about the beginning of the month, fell again considerably soon after, and continued very variable during the remainder.

The thermometer also varied considerably. During some of the pleasant days, it rose much higher than even at any time during last month; but, at other times, it fell very low, especially in the cold mornings and evenings.

The complaints mentioned last month still continue to prevail, with the addition of other diseases of a chronic nature, such as asthma and chronic catarrh. Some of the complaints of last month too, have, in many instances, assumed a greater degree of violence, and have occurred in practice, blended with each other. This has been particularly remarkable in the bowel complaints appearing blended with typhus fever. The fevers, even uncombined, are of a very bad kind. In various parts of the city, where the houses are closely pent up together, preventing that degree of circulation of air which is absolutely necessary to the preservation of health, these fevers are highly malignant and contagious. Many families, situate as described, have suffered severely from that disease.

The bowel complaints, which now indiscriminately affect old and young persons, continue with unabated severity, and, in many instances, they are accompanied by excruciating pain, and considerable discharges of blood, producing great debility in a very short period.

These two diseases, fever and bowel complaints, have frequently occurred in combination, and have in that form often terminated fatally.

These bowel complaints are but indifferently attended to. They are, as is the case with many other diseases, not immediately destructive in their consequences, which is one strong reason that they are often shamefully neglected.

I saw a case, some time ago, of an old lady, who had by an accident broken the tibia and fibula of her right leg. She then resided a few miles in the country. After replacing the bones, her surgeon, by way of saving the leg as much as possible from injury, seemed to think it improper to put her to trouble in evacuating her bowels, as he paid no attention to them for nearly seven weeks; during that period no evacuation of them took place oftener than twice. She was then seized with a violent bowel complaint, and her friends brought her to Edinburgh, and put her under my care. The bowel complaint, at this time, had ceased for two days, but there was great tension and and considerable inequality felt on applying the hand to the abdomen. I next day desired that Dr. Gregory should be called; and she being very stout, we endeavoured to evacuate her bowels by brisk cathartics and injections. We in part effected our purpose, but not to the extent we could have wished, or that even seemed necessary. Dr. Gregory then suggested the propriety of administering a warm water injection; and after friction had been applied to the abdomen, there was thrown up by the rectum about four pounds of warm water, but this occasioned no evacuation; in a few hours after the same quantity was repeated; still, however, no evacuation followed. Strong decoctions of senna and tamarinds were at the same time taken, but soon after our patient fell into a state of stupor and expired. The relations would not suffer the body to be opened; but there is no doubt that she died of mortification of the bowels, and this in consequence of the neglect of a man, who practices extensively, and is deemed a good surgeon in the country.

To those men who practice their profession, and who, for the first time, have been informed by Dr. Hamilton of this place, in his book on bowel complaints, that diarrhœa, for instance, may be caused by an accumulation of fæces in the intestinal canal, have been shamefully negligent; and those who, at this late period, require to be informed, that in such complaints, instead of the immense variety of astringent mixtures, &c. which they had been in the habit of using for the removal of such complaints, more permanent effects may be produced by a dose or two of calomel and jalap, ought to be ashamed at the necessity of such a publication.

The indiscriminate use of any medicine must be wrong, and the injury sustained from it must be in proportion to the extent of opportunity which the physician has of unnecessarily employing it.

The propriety of employing purgative medicines is not to be doubted, and their efficacy in the removal of disease is certainly very great; yet they may, unquestionably, have been, and are daily, used in this part of the world particularly of late, with rather too liberal a hand.

Among the first lessons I recollect to have received in physic, to regulate the natural evacuations, and to relieve, if possible, morbid discharges of every description, was one; I therefore did not understand that the useful observations of Dr. Hamilton, on that subject, could at all be classed among our modern discoveries. The enthusiasm, therefore therefore, which many felt on such a principle being announced by Dr. Hamilton, can only be explained in one way. That is, that such gentlemen as now use nothing else for the removal of almost *every* disease, had, previous to Dr. Hamilton's publication, paid no attention to the state of the bowels, and since that time no other consideration has been capable of attracting their attention. Whatever, therefore, the state or nature of the disease may be, they are determined to purge it off. Thus, by indiscriminately administering a useful medicine, while they show their own want of discrimination, they fail in effecting any useful purpose; and thus only bring on themselves disgrace, while they furnish a handle to those, who are anxious to find fault with every thing, to ascribe the failings of the physician solely to the general inutility of medicine.

It is indeed very plain, that Dr. Hamilton's book inculcates no more than what must have been known to every person who has studied enough of his profession to know, that when the bowels are overloaded with fæces, they must in the first instance be emptied with all imaginable speed.

Had the Doctor demonstrated to us, that purgatives possessed the power of curing complaints by certain specific qualities which could be attributed to them, independently of the action of the bowels being rendered regular, previous to their exhibition, he would certainly have been entitled to the praise of originality ; but he neither can, nor I suppose, intends to claim it in the present instance. The same may be said of Mr. Abernethy of London, who, after Dr. Hamilton, cures an infinite number of diseases by purgation.

But to return : I may observe, that, on the present occasion, purgative medicines seem entitled to a preference in the bowel complaints, which are so common among us. Under certain circumstances, however, drastic purgatives create most excruciating pain ; and I have, for the most part, found that a compound of equal parts of extracts of hyociamus and aloes, is preferable to any other purgative. While it is effectual in its operation, it produces no pain or even uneasiness. I have, in various instances, found it necessary to administer acid in a diluted state, in order to put a stop to the discharge of blood, which in some cases accompanied these bowel complaints. By these medicines I have experienced greater benefit than with any other.

Asthma

Asthma and chronic catarrh seem to have spread considerably since the commencement of the month. These complaints seem rather to admit only of temporary palliation than of complete removal.

From the long continuance of wet weather, we meet with many other complaints, but they are in general of a less decisive and less formidable nature than those I have already mentioned.

*Princes Street.*

